# The stability of the twofold multidimensionality of academic self-concept: A study of Chinese secondary school students

**DOI:** 10.3389/fpsyg.2022.1001187

**Published:** 2023-01-06

**Authors:** Feifei Han, Kateřina Juklová, Petr Mikoška, Lukáš Novák

**Affiliations:** ^1^Institute for Learning Sciences and Teacher Education, Australian Catholic University, Brisbane, QLD, Australia; ^2^Department of Pedagogy and Psychology, Faculty of Education, University of Hradec Králové, Hradec Králové, Czechia; ^3^Olomouc University Social Health Institute, Palacký University in Olomouc, Olomouc, Czechia

**Keywords:** academic self-concept, twofold multidimensionality, stability, Chinese secondary school students, internal/external frame of reference model

## Abstract

**Introduction:**

The present investigation examined the stability of the twofold multidimensional structure of academic self-concepts (ASCs) in three domains, namely Chinese, math, and general school using four-wave data collected over 2 years among 552 Chinese secondary school students.

**Method:**

Adopting both a within-network and a between-network approach, confirmatory factor analyses (CFAs) and factor correlations were performed in Mplus 8.2.

**Results:**

The within-network results showed that CFA models wherein competence and affect dimensions were conflated generated unacceptable fit. In contrast, the CFAs in which competence and affect were modeled as separate latent factors consistently produced superior fit to the data. The between-network results demonstrated that in the Chinese and math domains and across the four-time waves, the competence components were more strongly related to the achievements in matching domains than the affect components were. Furthermore, both the competence and affect components of ASCs and achievements were positively correlated in the non-matching domains, which were somewhat contradictory to the internal/external frame of reference model predicting zero or negative relations.

**Discussion:**

Such results seem to suggest more involvement in social comparison than in dimensional comparison of Chinese students, which might be attributed to the collectivistic Chinese culture and the common phenomenon of academic social comparisons among Chinese adolescents in schools.

## 1. Introduction

Defined as “a person’s perception of himself formed through his experience with his environment and influenced especially by environmental reinforcements and significant others” (Shavelson et al., 1976, 411), self-concept (SC) is widely acknowledged as an important psychological construct, vital in all parts of human life, and it has been researched in a variety of fields, including education, psychology, sport sciences, mental health, and many more (Marsh and Craven, 2006; Onetti-Onetti et al., 2019; Karimova and Csapó, 2021; Henning et al., 2022; Melguizo-Ibáñez et al., 2022). For decades, empirical research has shown that having positive SC is beneficial for many areas of human functioning (Marsh et al., 2017): fighting against anxiety and distress, for strengthening confidence and resilience, and for being adaptive to handle complex problems and situations (Coopersmith, 1960; Harter, 1990; Dodgson and Wood, 1998; Sommer and Baumeister, 2002), and thus enables many human potentials (Marsh and Craven, 2006; Marsh and O’Mara, 2008).

Among various forms of SC, students’ self-perceptions in school curriculum domains (e.g., general school, math, verbal, and science), known as academic self-concept (ASC), have been extensively researched. Research has been conducted to identify factors which may influence ACS and has shown that teacher relatedness (Guay et al., 2019), peer feedback (Simonsmeier et al., 2020), and socioeconomic status (Chevalère et al., 2022) can impact ACS. Furthermore, ASC has been found to be closely associated with various desirable academic outcomes, such as academic achievement, coursework selections, learning motivation and confidence, course and career aspirations, effort expenditure, and use of self-regulated learning strategies (Marsh and Craven, 2006; Marsh and Martin, 2011; Guo et al., 2015; Yang et al., 2016; Kadir et al., 2017; Schneider and Sparfeldt, 2020; Deeba et al., 2022; Maynor et al., 2022). In the past five decades, this knowledge has been constantly expanded and refined by new findings on the multidimensional nature of ASC (Marsh et al., 1999a); the reciprocal relationships between ASC and academic achievement (Marsh and Craven, 2006; Marsh and O’Mara, 2008); the domain-specific relations of ASC with academic achievement (Chiu and Klassen, 2010; Kadir et al., 2017; Arens and Preckel, 2018); and the effects of internal and external comparison processes in the formation of ASCs and explaining the interrelations of ASCs in different domains (Möller et al., 2009; Möller and Marsh, 2013).

An equally important finding is the validity of the twofold structure of ASC, which conceptualizes ASC as being formed by distinct cognitive (based on the student’s self-perceptions of the competence in a domain) and affective (based on the student’s feelings toward a relevant domain) factors (Marsh et al., 1999b). Past research has demonstrated that ASC has both multidimensional and twofold characteristics, known as “the twofold multidimensional structure of ASC” (Rosenberg, 1979; Arens et al., 2011; Gorges and Hollmann, 2019; Smith, 2019). However, most of these studies, whether cross-sectional or longitudinal, either only investigated the twofold structure of ASC in a single domain or examined it across more domains, but at one time point, hence failed to test the stability of the multidimensional and twofold structure simultaneously. The present study aims to fill this gap by investigating the stability of the twofold multidimensional structure of ASC in three domains—general school, verbal, and math—among Chinese secondary students using three-wave longitudinal data.

## 2. Theoretical framework

### 2.1. Theoretical bases of twofold multidimensional ASC

Since the breakthrough in SC research by Shavelson et al.’s review (Shavelson et al., 1976), which refuted the unidimensional conceptualization of SC (Rosenberg, 1979; Marx and Winne, 1978), modern theory has emphasized SC as a multidimensional construct (Arens et al., 2011). While Shavelson et al. proposed a global SC which is formed by a nonacademic SC and an ASC factor, confirmatory factor analyses (CFAs) could not identify a global ASC with students in different age groups, including preadolescents (Marsh, 1990c), adolescents (Marsh, 1990b), and late adolescents and young adults (Marsh and O’Neill, 1984). This resulted in the Marsh/Shavelson model of ASC (Marsh, 1990d), which conceptualizes students’ ASC as being represented by two distinct higher-order factors of verbal and math ASC. Students’ ASCs in various curriculum domains are conceptualized as first-order factors on a continuum, with verbal and math ASCs on the two ends of the continuum and school ASC is in the middle. Therefore, at the one end of the continuum, the first-order verbal ASC has exclusive loadings on the second-order verbal ASC; at the other end of the continuum, the first-order math ASC has exclusive loadings on the second-order math ASC; and in the middle of the continuum, the first-order school ASC has loadings on both the second-order verbal and math ASCs. Such conceptualization has received support from many empirical studies which identified distinct ASCs in various curriculum domains, including general school, verbal, math, physics, art, music, history, and many other school subjects (Marsh et al., 1988; Marsh, 1990a; Arens et al., 2011).

In addition to its multidimensionality, another feature of ASC is its twofold structure, which further makes a distinction between the competence (the cognitive aspect of SC) and affect (the affective aspect of SC) components within each curriculum domain (Marsh et al., 1999b; Arens et al., 2011, 2014b, 2016). Although the competence and affect ASC has been treated as a single factor for some time, the twofold structure of ASC has a sound theoretical basis. For instance, Irwing (1996) provided some theoretical arguments as to why competence and affect should be distinguished. He maintains that essentially an individual tends to adopt different benchmarks when evaluating levels of capabilities and affective status. When judging one’s own competence, a person tends to use others’ qualities as a reference point whereas when evaluating one’s own affect, an internal comparison of feelings between one domain and another domain is more likely. In line with Irwing’s arguments are the assumptions of the internal/external frame of reference model (I/E model) postulated by Marsh (1986), who offers reasons for different relations between ASCs and academic achievements within and across domains. The I/E model similarly assumes the operations of both internal and external comparison, through which the domain-specific ASCs are formed. In an updated I/E model, Möller and Marsh (2013) proposed a dimensional comparison model which considers dimensional (similar to internal) and social (similar to external) comparison as mechanisms establishing interrelations between ASCs and achievements both within and across domains. Both the I/E and dimensional comparison models posit that internal (dimensional) comparison is based on comparing one’s own abilities in one domain with own abilities in other domains, whereas external (social) comparison considers an individual’s own ability against the perceived ability of his/her classmates. Given the operation of both internal (dimensional) and external (social) comparisons in the processes of forming domain-specific ASCs, despite the high positive correlations among academic achievements across domains, ASCs in dissimilar domains are uncorrelated. Furthermore, compared to significant positive within-domain relations between ASCs and achievements, zero, near zero or negative cross-domain correlations between achievement and academic self-concept are expected (Marsh, 1986, 2014; Marsh et al., 2012; Möller and Marsh, 2013).

Distinguishing the cognitive and affective ASC components and simultaneously assuming that social comparison is more involved in assessing the cognitive (competence) component and dimensional comparison is more present in evaluating the affective (affect) component, it can be expected that the relations between the two components of ASCs and achievements in the corresponding domains will differ from those across academic domains. In particular, while achievement will have positive associations with both competence and affect within a domain, it is expected that the strength of the association between affect and achievement should be weaker than that between competence and achievement, as more dimensional comparison takes place in evaluating affect. Across domains, correlations between the two components of ASC and academic achievement can be expected to be near zero, zero, or negative, and such correlations will also be weaker for affect and achievement than for competence and achievement.

### 2.2. The within-network approach to testing the twofold multidimensionality of ASC

The co-existence of the multidimensional and twofold nature of ASC is known as “the twofold multidimensional ASC” (Arens et al., 2011; Yang et al., 2016). In the past two decades, many studies have been conducted to empirically test the twofold multidimensionality of ASC adopting both a within-network and a between-network approach. According to Arens et al. (2011), the within-network approach focuses on evaluating the validity of internal structure of ASC by adopting the exploratory and/or confirmatory factor analyses (CFAs), whereas the between-network approach emphasizes investigating the extent to which the constructs of ASC and other external criteria (constructs) overlap. Hence, the within-network approach examines the internal validity of ASC, whereas the between-network approach investigates the external validity of ASC. Researchers strongly recommend including both the within-network and the between-network approaches for rigorous testing of the construct validity of ASC (Shavelson et al., 1976; Byrne, 1984, 1996; Arens et al., 2011; Arens and Preckel, 2018).

Studies adopting the within-network approach have examined twofold multidimensional ASC with a diverse population. The general findings of these studies provided empirical evidence that the twofold multidimensional structure of ASC is generalizable to students at different stages of schooling, including students in preschools (Arens et al., 2016), primary schools (Marsh et al., 1999b; Arens and Hasselhorn, 2015; Arens and Morin, 2016; Arens and Preckel, 2018; Lohbeck, 2019; Schneider and Sparfeldt, 2020), secondary schools (Marsh et al., 1999b, 2013; Arens et al., 2014a; Kadir et al., 2017), vocational schools (Yang et al., 2016), and universities (Burns et al., 2018). Research has also been carried out to examine if the twofold multidimensional structure of ASCs is also applicable to students from diverse cultures and has found support among students from Western cultures (e.g., German: Arens et al., 2011; Lohbeck and Möller, 2017; Lohbeck, 2019; Schneider and Sparfeldt, 2020; Dutch: Pinxten et al., 2013; French-Canadian: Marsh and Ayotte, 2003; Anglo-Saxon: Marsh et al., 1999b); from Arabic cultures (e.g., Abu-Hilal et al., 2013; Marsh et al., 2013); from Indigenous cultures (e.g., Indigenous Australian: Arens et al., 2014a); and from Eastern countries (e.g., China: Yang et al., 2016; Leung, 2019).

### 2.3. The between-network approach to testing the twofold multidimensionality of ASC

Not all the studies adopting a within-network approach also include a between-network approach to testing the twofold multidimensionality of ASC. Those studies including the between-network approach also provide some evidence for the validity of the twofold multidimensionality of ASC. Of the different types of the external criteria, the short-term educational outcome—academic achievement—has been the one most frequently used. Studies have reported that consistently in various academic domains, the competence and affect components of ASC have different relations with academic achievement among students at different stages of schooling and in various school domains, including students in primary schools (Arens et al., 2011: general school, verbal, and math; Arens and Preckel, 2018: verbal, math, and physical education; Lohbeck, 2019: verbal and math; Schneider and Sparfeldt, 2020: verbal and math) and in secondary schools (Abu-Hilal et al., 2013: math and science; Arens et al., 2014b: verbal and math; Kadir et al., 2017: verbal, math, and physics; Marsh et al., 2013: math and science; Karimova and Csapó, 2021: verbal and foreign language).

For instance, with German primary school students, Arens et al. (2011) found that consistently across three domains, namely general school, German, and math, the correlations between competence and achievement were doubled those between affect and achievement in the matching domains. Such results were corroborated by the results in Abu-Hilal et al. (2013) with 2,687 eighth-grade Saudi students in math and science domains that the associations between competence and achievement were more than triple those between affect and achievement in the matching domains. However, such patterns of the different relations between competence-affect and learning outcomes were not found when the external criteria were course aspirations and effort, which were considered as being longer-term learning outcomes. As argued by Yeung et al. (2012) that students’ high sense of competence is more likely to have positive relations with the assessment-based outcomes, such as GPAs and test results, students´ strong liking tends to be positively associated with longer-term outcomes, such as persistence and motivation in learning the subject.

For example, Marsh et al. (2013) reported that among 8th graders, the relations between affect and course aspirations in the matching domain were more strongly correlated than those between competence and course aspirations in the matching domains. Such results were reasonably consistent across math and science domains and across two cultures (Anglo-Saxon culture: students from the United States, England, Australia, and Scotland and Arab cultures: students from Saudi Arabia, Jordan, Oman, and Egypt).

Using effort as an external criterion, however, the relational patterns are not consistent. With German primary school students, Arens and Hasselhorn (2015) found that consistently in both verbal and math domains, the correlations between affect and effort were stronger than those between competence and effort in the matching domains. Also with German primary school students, however, Lohbeck (2019) reported that the correlations between affect and effort in the corresponding domains were similar to those between competence and effort in the corresponding domains. The two studies adopted different ways of measuring students’ perceptions of effort. While Arens and Hasselhorn (2015) measured effort in a general sense, Lohbeck (2019) assessed effort in domain-specific ways. Whether such difference contributed to the inconsistent results found in the two studies requires further investigations in future studies.

Whether adopting within-network and/or between-network approaches, the majority of existing studies are cross-sectional, and, therefore, despite support for the twofold multidimensional structure of ASC, they were not able to test whether such structure was stable across time. Essentially, longitudinal studies are needed in order to show whether the twofold multidimensional structure of ASC can be clearly established over time.

### 2.4. The stability of twofold multidimensional ASC

To date, little research has been carried out to examine the stability (longitudinal design) of the twofold multidimensional structure of ASC. The longitudinal design which examined the validity of separating the competence and affect aspects of ASC have predominantly been held in a single domain (general school: Han, 2019; math: Pinxten et al., 2014; Arens et al., 2016; psychology: Burns et al., 2018). While these studies confirmed the stability of separating competence and affect in these specific domains, it failed to address the twofold structure and multidimensional structure of ASC simultaneously.

Adopting both a within- and a between-network approach, Pinxten et al. (2014) collected five-wave data to examine: (1) the structure of Dutch primary school students’ math ASC in terms of a single factor conflating competence and affect or a clear distinction between competence and affect and (2) the relations between math ASC (competence-affect) on the one hand and math achievement and math effort expenditure on the other hand. The results of the within-network analysis demonstrated that a two-factor model (separating competence and affect) was superior to a one-factor model (conflating both competence and affect components). The results of the between-network approach showed that the relations between competence-affect and achievement had reversed pattern with the relations between competence-affect and effort. Specifically, reasonably across time, the relations between competence and achievement were stronger than those between affect and achievement. In contrast, the associations between competence and effort were weaker than those between affect and effort. The study provided support for the stability of the twofold structure of math self-concept and the stability of the relations between competence-affect and short-term (i.e., achievement) and long-term (i.e., effort) educational outcomes in math domain.

In terms of investigation of the stability of ASC in multiple domains, Marsh et al.’s (1999b) study is the only one that examined whether separating competence and affect was stable across a number of domains. As the participants in Marsh et al. are Australian primary school students, there is a lack of investigation of the stability of the twofold multidimensionality of ASC with older students (e.g., secondary school students) or in other cultures (e.g., Eastern cultures). Another limitation of Marsh et al.’s study was that it did not examine if competence-affect had different relations with academic outcomes (e.g., academic achievement), while cross-sectional studies in multiple domains have reported that the competence ASC had a higher correlation with academic achievement than the affect ASC not only within the matching domain but also across domains (Arens et al., 2011). The longitudinal studies in a single domain also reported that the different relations between competence-affect and academic achievement were consistent across time waves (Pinxten et al., 2013; Arens et al., 2016; Han, 2019). However, the single-domain longitudinal studies failed to investigate the longitudinal patterns of competence-affect and academic achievement across domains.

### 2.5. Present investigation

To fill the above-mentioned gaps in the literature, the aims of the present investigation were twofold. First, we aimed to extend Marsh et al. (1999a) examination of the stability of the twofold multidimensional ASC to a much less researched population—students in mainland China. As the students in Marsh et al. were primary school students, we would target an older population—secondary school students. Second, Marsh et al.’s study did not include students’ academic achievement and thus it was not able to comprehensively test the construct validity of ASC by including external criteria. While both cross-sectional and longitudinal studies reported that competence had stronger association with achievement than affect did in the matching domain, the present study would investigate whether the differential associations between competence and affect in relation to academic achievement in the matching domain were consistent across domains and across time among Chinese secondary school students. To fulfill the two research aims, the present study aimed to investigate the stability of the twofold multidimensionality of ASC with Chinese secondary school students in the general school, verbal and math domains in a four-wave dataset collected over 2 years by adopting both a within-network and a between-network approach.

According to the literature review, the following three hypotheses were formulated, one for the within-network approach, and two for the between-network approach:

*H1*: Across four waves, Chinese secondary school students’ ASCs would consist of competence and affect factors rather than a single factor conflating competence and affect in all the three domains (the within-network approach).

*H2*: Across four waves, self-perceptions of competence would be more strongly associated with achievements than self-perceptions of affect within both the Chinese and math domains (the within-network approach).

*H3*: Across four waves, the relations between competence and affect components and achievements would be zero or negative in non-matching domains.

## 3. Materials and methods

### 3.1. Participants and data collection procedure

The study was conducted in a junior secondary school in mainland China. The reason for using one school was all the students would sit the same examinations, allowing their academic performance to be compared. The recruitment of the participants targeted grade 7 students (the first year of junior secondary school). Because the study was designed as a longitudinal study, lasting for 2 years. Chinese junior students in the last year of junior secondary school face pressure of attending the high school matriculation examination. This meant that when students would complete the last wave of data collection, they would be in grade 8. There were 630 students in grade 7 and all of them were invited to participate on a voluntary basis. Altogether 552 students (268 girls: 48.6%; and 284 boys: 51.4%) participated in the study, accounting for 87.6% of the total population.

Ethical procedures were strictly followed. Before the data collection, written consent was obtained from all the participants and their parents. T1 data collection was done approximately toward the end of the first term in grade 7, and T2 data collection was done approximately toward the end of the second term in grade 7. T3 and T4 data collection was done toward the end of the first and second terms, respectively, in grade 8. The ages of the participants were approximately between 13 and 15 years old during the first round of data collection (T1) and were between 15 and 17 years old during the last round of the data collection (T4). The vast majority of students stayed for the whole duration of 3 years of junior high school; therefore, missing data amounted to only 6.7%. To deal with this missing data, we adopted the full information maximum likelihood (FIML) estimation in Mplus. According to Graham (2009), full FIML is generally considered to be a better approach to dealing with missing data than the traditional approaches, such as listwise deletion and pairwise deletion.

### 3.2. Measures

#### 3.2.1. ASC

We used the Chinese version of SDQ I to measure the participants’ ASC in general school, math, and Chinese. The psychometric properties of the Chinese version of SDQ I were validated with Chinese primary and secondary students in mainland China (Watkins et al., 1997). As our participants were secondary school students in mainland China, it was considered appropriate to use this instrument. We used all the original items for the general school ASC and math ASC scales in the Chinese version of SDQ I, which corresponded with the items in the original English SDQ I. However, we modified the items in the reading ASC scale as past research has suggested that the reading ASC was only a sub-facet of the verbal ASC and represents specific sets of verbal ASC skills (Yeung et al., 2012; Arens et al., 2014b). Thus, the word “reading” in the reading ASC in the Chinese version of SDQ I was replaced by the word “Chinese” to present students’ verbal ASC in their native language. For instance, the item “I am good at reading” was changed into “I am good at Chinese.”

There were 10 for each domain and the wording of items was paralleled in the three domains. In each domain, five items captured students’ self-perceptions of capability in terms of their performance (i.e., the cognitive aspect of ASC), and another five items assessed students’ self-appraisal of their enjoyment and liking of the subject (i.e., the affective aspect of ASC). Of the five items, four items were positively worded and one was negatively worded item, which was useful as the negatively worded items helped tease out any possible response bias (Arens et al., 2011). All the items were placed on a 5-point Likert scale, with the anchors representing the following: 1 = false, 2 = mostly false, 3 = sometimes false, sometimes true, 4 = mostly true, and 5 = true.

#### 3.2.2. Academic achievement

As the students were from the same school and the same grade, we used students’ performance in the final-term Chinese and math exams as indicators of their academic achievement. Due to the strict policies in the school, the exams had high standards and all the students were required to take part in the exams. We obtained their scores directly from the school’s administrative system.

### 3.3. Data analysis

Data analyses were conducted using the within-network approach and the between-network approach, both of which performed a series of CFAs, which aim to test pre-specified models based on theory and previous literature (Brown, 2015). Mplus version 8.2 was used to analyze the data. We followed the general procedures proposed by Kline (2015) and Joreskog and Sorbom (2005) to evaluate the fit of CFAs, and considered the following goodness-of-fit indices as primary indicators of model fit: the Tucker-Lewis Index (TLI) (Tucker and Lewis, 1973), the Comparative Fit Index (CFI) (Bentler, 1990), and the root mean square error of approximation (RMSEA) (Browne and Cudeck, 1992). According to Bentler (1990) and Hu and Bentler (1999), a TLI or CFI value higher than 0.90 is generally considered an acceptable fit to the data. With regard to the RMSEA value, Browne and Cudeck (1992) suggest that RMSEA values below.06 are indicative of a good fit between the hypothesized model and the observed data. In addition to these fit statistics, we also consulted the two other criteria recommended by Joreskog and Sorbom (2005) in order to evaluate the appropriateness of the models. The first criterion was that the factor loadings of items in corresponding scales should be greater than 0.30. Secondly, the correlations between the two factors should be smaller than.90 in order to ensure the two scales are clearly distinguishable from each other.

#### 3.3.1. Within-network approach to CFAs

The within-network approach to CFA tests the internal structure of ASC. We first constructed two models separately for each domain: models 1 and 2 for ASC in Chinese, models 3 and 4 for ASC in math, and models 5 and 6 for ASC in general school. In each domain, the first models (models 1, 3, and 5) were one-factor CFAs of ASC, in which all the ten items were conflated to represent a single latent ASC factor. The second models in each domain (models 2, 4, and 6) were two-factor CFAs of ASC, with five items forming a competence factor of competence and the other five items forming an affect factor.

Following these separate CFAs in each domain, we then constructed CFAs for the three domains together in order to examine the twofold multidimensional structure of ASC. Model 7 comprised three separate factors for ASCs in Chinese, math, and general school respectively, but ignored the distinction between competence and affect in each domain, whereas model 8 was established to form a six-factor model which consisted of three domains and separated competence and affect in each domain. For all the models, the error terms of the same items were correlated as each item was used repeatedly for each wave of data collection. In addition, we also included correlated uniquenesses in models 7 and 8, as parallel worded items were used across Chinese, math, and the general school domains.

#### 3.3.2. Measurement invariance

Before conducting between-network approaches to CFAs, we also conducted measurement invariance test to examine if the latent construct stayed the same over time (Möller et al., 2011). The invariance tests were conducted by increasingly constraining parts of the measurement structure. We followed Brown’s (2015) recommended procedure for performing the invariance tests in a stepwise manner from loose to tight. The invariance tests involved evaluating three levels of restricted models: (1) a configural model (10A), which tested whether the factor structures were identical across years. (2) the metric model (10B), which tested whether the factor loadings were equal; and (3) the scalar model (10C), which tested whether the intercepts were equal. For model comparison, we adopted the following guidelines: a decrease in fit of less than 0.010 in CFI (Cheung and Rensvold, 2002; Chen, 2007) and an increase in fit of less than 0.015 in RMSEA (Chen, 2007) would support the more parsimonious model.

#### 3.3.3. Between-network approaches to CFAs

For between-network approaches, we integrated academic achievement in Chinese and math as external validity criteria into the three final retained CFA models in the within-network approaches. Similar to the procedure adopted in our within-network approach analyses, we also constructed separate CFAs for each domain first (model 11 for Chinese and model 12 for math respectively). If the one-factor models (10 items conflating competence and affect) had a better fit, we would add achievement into the one-factor models, and if the two-factor models (5 items representing competence and 5 items representing affect) produced better fit, we would add achievement to the two-factor models. When Chinese, math, and general school considered together (model 13), if the three-factor model had a better fit, we would add Chinese and math achievement results into the three-factor model; and if the six-factor model had a better fit, we would add Chinese and math achievement results into the six-factor model. As the achievement scores were single-item indicators, the measurement error of scores was fixed as 0.95.

## 4. Results

### 4.1. Results of the within-network approach to CFAs

Models 1 to 10 represented the within-network approach to CFAs and their results are displayed in the upper half of Table 1, which summarizes the values of Chi-square, degree of freedom, and the fit statistics of the within-network CFA models. The results of the CFAs conducted for the three domains separately were as follows: Chinese: model 1: *χ*^2^ (674) = 2927.83, CFI = 0.83, TLI = 0.81, RMSEA = 0.07; math: model 3: *χ*^2^ (674) = 2788.58, CFI = 0.87, TLI = 0.85, RMSEA = 0.07; general school: model 5: *χ*^2^ (674) = 3629.01, CFI = 0.78, TLI = 0.75, RMSEA = 0.08); and these were the results for the three domains simultaneously: model 7: *χ*^2^ (6654) = 16229.77, CFI = 0.82, TLI = 0.81, RMSEA = 0.06. All showed that the one-factor CFIA models of ASC failed to yield acceptable fit, even when the negatively worded items were deleted (model 8: *χ*^2^ (4158) = 10360.87, CFI = 0.86, TLI = 0.85, RMSEA = 0.05).

**Table 1:** Goodness of fit of CFA models.

Within-network approach						
Model	Model description	*χ* ^2^	*df*	CFI	TLI	RMSEA
1	One-factor CFA model for Chinese	2927.83	674	0.83	0.81	0.07
2	Two-factor CFA model for Chinese	1374.77	652	0.95	0.94	0.04
3	One-factor CFA model for math	2788.58	674	0.87	0.85	0.07
4	Two-factor CFA model for math	1217.13	652	0.97	0.96	0.04
5	One-factor CFA model for general	3629.01	674	0.78	0.75	0.08
6	Two-factor CFA model for general	1549.45	652	0.93	0.92	0.05
7	Three-factor CFA model for Chinese, math, and general (correlated)	16229.77	6,654	0.82	0.81	0.06
8	Three-factor CFA model for Chinese, math, and general (correlated, elimination of negative worded items)	10360.87	4,158	0.86	0.85	0.05
9	Six-factor CFA model for Chinese, math, and general (correlated)	10865.93	6,444	0.92	0.91	0.03
10	Six-factor CFA model for Chinese, math, and general (correlated, elimination of negative worded items)	6283.48	3,948	0.95	0.94	0.03
Between-network approach						
11	Two-factor CFA model for Chinese + Chinese achievement	1541.41	780	0.95	0.94	0.04
12	Two-factor CFA model for math + math achievement	1513.13	780	0.96	0.95	0.04
13	Integration of Chinese and math achievements into model 10	7159.90	4,524	0.95	0.94	0.03

CFI, comparative fit index; TLI, Tucker-Lewis index; RMSEA, root mean square error of approximation.

In contrast, the two-factor CFAs of self-concept wherein competence and affect represented distinct latent factors generated good fit when Chinese, math, and general school were examined separately (Chinese: model 2: *χ*^2^ (652) = 1374.77, CFI = 0.95, TLI = 0.94, RMSEA = 0.04; math: model 4: *χ*^2^ (652) = 1217.13, CFI = 0.97, TLI = 0.96, RMSEA = 0.04; general: model 6: *χ*^2^ (652) = 1549.95, CFI = 0.93, TLI = 0.92, RMSEA = 0.05); and when the three domains were jointly considered (model 9: *χ*^2^ (6444) = 10865.93, CFI = 0.92, TLI = 0.91, RMSEA = 0.03). On the basis of model 9, we then eliminated negatively worded items of each domain to construct model 10, and the fit statistics showed even better fit (model 10: *χ*^2^ (3948) = 6283.48, CFI = 0.95, TLI = 0.94, RMSEA = 0.03).

Apart from the fit statistics, we also checked the factor loadings in these models, and the results show that the loadings of items for their corresponding factors were all above 0.65. As model 10 which tested the twofold multidimensional structure of ASC in four-time waves by eliminating negatively worded items produced better fit, we used model 12 to calculate the factor correlations, which were all below 0.86. Furthermore, we also calculated Cronbach’s alpha coefficients for all the competence and affect scales in the three domains across four-time waves, which showed that all the values had fairly good reliability, as all the αs were above 0.70 (see Table 2). The descriptive statistics, including *M*s and *SD*s of competence and affect in the three domains across four-time waves are also displayed in Table 2. The factor loadings, factor correlations, and values of scale reliability together with the fit statistics demonstrated that the twofold multidimensional structure of ASC in the Chinese, math, and general school domains was stable across 3 years for Chinese secondary school students.

**Table 2:** Descriptive statistics and reliability of scales.

	ChiCOM	ChiAFF	MatCOM	MatAFF	GeCOM	GeAFF
	*M* (*SD*) alpha	*M* (*SD*) alpha	*M* (*SD*) alpha	*M* (*SD*) alpha	*M* (*SD*) alpha	*M* (*SD*) alpha
T1	3.31 (0.70) 0.88	3.75 (0.79) 0.88	3.46 (0.75) 0.87	3.87 (0.79) 0.82	3.33 (0.61) 0.80	3.91 (0.73) 0.77
T2	3.36 (0.72) 0.90	3.90 (0.77) 0.81	3.50 (0.82) 0.92	4.01 (0.77) 0.83	3.34 (0.69) 0.87	3.99 (0.78) 0.82
T3	3.32 (0.73) 0.89	3.85 (0.79) 0.85	3.44 (0.83) 0.92	3.93 (0.82) 0.84	3.31 (0.75) 0.89	3.92 (0.83) 0.84
T4	3.34 (0.74) 0.87	3.65 (0.86) 0.81	3.39 (0.86) 0.91	3.83 (0.87) 0.94	3.21 (0.84) 0.83	3.69 (1.00) 0.83

ChiCOM, Chinese competence scale; ChiAFF, Chinese affect scale; MatCOM, math competence scale; MatAFF, math affect scale; GeCOM, general school competence scale; GeAFF, general school affect scale.

### 4.2. Results of measurement invariance tests

As model 10 produced a better fit, we thus examined the twofold multidimensional structure of ASC in four-time waves without negative items. The results of the invariance tests are presented in Table 3. Our configural model (without constraining of factor loadings) produced good fit: χ^2^ (4092) = 1549.95, CFI = 0.933, TLI = 0.925, RMSEA = 0.034. Our metric model which constrained all factor loadings of the same latent construct to be equal over time resulted in a non-substantial decline in model fit: χ^2^ (4146) = 7180.903, CFI = 0.932, TLI = 0.925, RMSEA = 0.034. The results suggest that the factor loadings of the twofold multidimensional structure of ASC, which separated competence and affect in Chinese, math, and general school, were equal over four-time points. Similarly, our scalar model also had a non-substantial decline in model fit: χ^2^ (4200) = 7485.158, CFI = 0.927, TLI = 0.920, RMSEA = 0.035, supporting the invariance intercepts across time.

**Table 3:** Results of the measurement invariance tests of model 10.

model	model description	*χ*^2^ (*df*)	CFI	∆CFI	TLI	RMSEA	∆RMSEA
10A	configural	1549.95 (4029)	0.933	---	0.925	0.034	---
10B	metric	7180.903 (4146)	0.932	−0.001	0.925	0.034	0.000
10C	scalar	7485.158 (4200)	0.927	−0.006	0.920	0.035	+0.001

### 4.3. Results of correlations between competence and affect

The factor correlations are displayed in Tables 4–7 for T1–T4, respectively. In general, all the cross-sectional correlations between competence and affect in each domain were positive. However, competence and affect were more highly correlated in Chinese (T1-T4: *r*s = 0.57, 0.62, 0.59, and 0.56) and math (T1-T4: *r*s = 0.60, 0.63, 0.56, and 0.64) than in general school (T1-T4: *r*s = 0.38, 0.42, 0.42, and 0.37).

**Table 4:** Factor correlations at T1.

	ChiCOM	ChiAFF	MatCOM	MatAFF	GeCOM	GeAFF	ChiACH
ChiAFF	0.57**	---	---	---	---	---	---
MatCOM	0.17**	0.05	---	---	---	---	---
MatAFF	0.14**	0.32**	0.60**	---	---	---	---
GeCOM	0.54**	0.39**	0.58**	0.39**	---	---	---
GeAFF	0.35**	0.63**	0.28**	0.57**	0.38**	---	---
ChiACH	0.30**	0.10*	0.11*	0.09*	0.27**	0.10*	---
MatACH	−0.04	−0.11*	0.52**	0.33**	26**	0.11*	0.40**

ChiCOM, Chinese competence scale; ChiAFF, Chinese affect scale; MatCOM, math competence scale; MatAFF, math affect scale; GeCOM, general school competence scale; GeAFF, general school affect scale; ChiACH, Chinese achievement; MatACH, math achievement. *p < 0.05, **p < 0.01.

**Table 5:** Factor correlations at T2.

	ChiCOM	ChiAFF	MatCOM	MatAFF	GeCOM	GeAFF	ChiACH
ChiAFF	0.62**	---	---	---	---	---	---
MatCOM	0.25**	0.11**	---	---	---	---	---
MatAFF	0.23**	0.42**	0.63**	---	---	---	---
GeCOM	0.65**	0.39**	0.61**	0.44**	---	---	---
GeAFF	0.46**	0.71**	0.29**	0.59**	0.42**	---	---
ChiACH	0.41**	0.16**	0.20**	0.10*	0.36**	0.12**	---
MatACH	0.09*	−0.02	0.57**	0.30**	0.37**	0.09*	0.60**

ChiCOM, Chinese competence scale; ChiAFF, Chinese affect scale; MatCOM, math competence scale; MatAFF, math affect scale; GeCOM, general school competence scale; GeAFF, general school affect scale; ChiACH, Chinese achievement; MatACH, math achievement. *p < 0.05, **p < 0.01.

**Table 6:** Factor correlations at T3.

	ChiCOM	ChiAFF	MatCOM	MatAFF	GeCOM	GeAFF	ChiACH
ChiAFF	0.59**	---	---	---	---	---	---
MatCOM	0.30**	0.16**	---	---	---	---	---
MatAFF	0.19**	0.50**	0.56**	---	---	---	---
GeCOM	0.69**	0.42**	0.67**	0.41**	---	---	---
GeAFF	0.38**	0.71**	0.33**	0.66**	0.42**	---	---
ChiACH	0.30**	0.09*	0.19**	0.10*	0.26**	0.08	---
MatACH	0.16**	0.07	0.43**	0.29**	0.34**	0.17**	0.62**

ChiCOM, Chinese competence scale; ChiAFF, Chinese affect scale; MatCOM, math competence scale; MatAFF, math affect scale; GeCOM, general school competence scale; GeAFF, general school affect scale; ChiACH, Chinese achievement; MatACH, math achievement. *p < 0.05, **p < 0.01.

**Table 7:** Factor correlations at T4.

	ChiCOM	ChiAFF	MatCOM	MatAFF	GeCOM	GeAFF	ChiACH
ChiAFF	0.56**	---	---	---	---	---	---
MatCOM	0.31**	0.19**	---	---	---	---	---
MatAFF	0.27**	0.48**	0.64**	---	---	---	---
GeCOM	0.67**	0.37**	0.62**	0.40**	---	---	---
GeAFF	0.40**	0.68**	0.36**	0.66**	0.37**	---	---
ChiACH	0.36**	0.16**	0.14**	0.07	0.30**	0.08	---
MatACH	0.13**	0.06	0.45**	0.25**	0.34**	0.09*	0.57**

ChiCOM, Chinese competence scale; ChiAFF, Chinese affect scale; MatCOM, math competence scale; MatAFF, math affect scale; GeCOM, general school competence scale; GeAFF, general school affect scale; ChiACH, Chinese achievement; MatACH, math achievement. *p < 0.05, **p < 0.01.

We also compared the relations of competence and affect across domains. For the competence aspect, we observed that consistently across four-time waves, the strength of the correlations between general school competence and Chinese competence and math competence were much stronger (general school and Chinese: *r*s = 0.64, 0.65, 0.69, and 0.67; general school and math: *r*s = 0.58, 0.61, 0.67, and 0.62) than those between Chinese competence and math competence (*r*s = 0.17, 0.25, 0.30, and 0.31). Likewise, we found stronger associations between general school affect and Chinese affect and general school affect and math affect (general school and Chinese: *r*s = 0.63, 0.71, 0.71, and 0.68; general school and math: *r*s = 0.57, 0.59, 0.66, and 0.66) than the association between Chinese affect and math affect (*r*s = 0.32, 0.42, 0.50, and 0.48).

Comparing the correlations between Chinese and math competence and those between Chinese and math affect, we found that one’s liking of Chinese and math are more related than one’s perceptions of ability in Chinese and math. Moreover, from a developmental perspective, the correlations also displayed an increase between Chinese and math competence and between Chinese and math affect, suggesting that feeling competent in the two subjects and feeling enjoyment while learning them became more related in grade 8 than in grade 7.

### 4.4. The results of the between-network approach to CFAs

Because the within-network approach to CFAs showed better fit results when competence and affect were considered separately, we established our between-network CFAs on the basis of the two-factor ASC. Similar to the within-network approach to CFAs, we also applied the between-network approach to CFAs separately for each domain first. On the basis of model 2, we constructed model 11 by adding Chinese achievement scores in three waves. Similarly, on the basis of model 4, we constructed model 12 by adding math achievement scores in three waves. Finally, on the basis of model 10, we constructed model 13 by adding both Chinese and math achievement scores in four waves. The results showed that the three models all produced good fit: model 11: *χ*^2^ (780) = 1541.41, TLI = 0.95, TLI = 0.94, and RMSEA = 0.04; model 12: *χ*^2^ (780) = 1513.13, TLI = 0.96, TLI = 0.95, and RMSEA = 0.04; and model 13: *χ*^2^ (4524) = 7159.90, TLI = 0.95, TLI = 0.94, and RMSEA = 0.03. These results demonstrated that the stability of the twofold multidimensional structure of ASC was also valid when applying the between-network approach.

The factor correlations between competence/affect and the academic achievement are presented in Tables 3–6, which showed that in both the Chinese and math domains, the correlations between competence and achievement (Chinese T1–T4: *r*s = 0.30, 0.41, 0.30 and 0.30; and math T1 to T4: *r*s = 0.52, 0.57, 0.43, and 0.45) were higher than those between affect and achievement (Chinese T1–T4: *r*s = 0.10, 0.16, 0.09, and 0.16; math T1–T4: *r*s = 0.32, 0.30, 0.29, and 0.25). These results indicated that self-evaluation of competence is more closely associated with one’s cognitive performance than the self-appraisal of affect in the matching domain.

Across domains, the results showed that the achievement scores had much weaker association with perceptions of competence and affect in non-matching domains. Chinese achievement had either weak or non-significant correlations with math competence and math affect in the four-time waves (Chinese achievement and math competence T1 to T4: *r*s = 0.11, 0.20, 0.19, and 0.14; Chinese achievement and math affect T1 to T4: *r*s = 0.09, 0.10, 0.10, and 0.07). Similarly, the associations between math achievement and Chinese competence were non-significant for T1 (*r* = −0.04, *p* = 0.43) and weak but positive for T2 to T4 (*r*s = 0.09, 0.16, and 0.13). The correlations between math achievement and Chinese affect were negative for T1 (*r* = −0.11) and non-significant for T2 to T4 (*r* = −0.02, *p* = 0.73; *r* = 0.07, *p* = 0.09; *r* = 0.06, *p* = 0.14). The relations between the achievement scores in Chinese and math and general school competence and affect appeared to be weaker compared to those of achievement and competence and affect in the matching domains (Chinese achievement and general school competence T1–T4: *r*s = 0.27, 0.36, 0.26, and 0.36; Chinese achievement and general school affect T1–T4: *r*s = 0.10, 0.12, 0.08 (*p* = 0.06), and 0.08 (*p* = 0.06); math achievement and general school competence T1 to T4: *r*s = 0.26, 0.37, 0.34 and 0.33; math achievement and general school affect T1–T4: *r*s = 0.11, 0.09, 0.17, and 0.09).

## 5. Discussion

The present study investigated the stability of the twofold multidimensionality ASC construct in three domains, namely Chinese and math, and general school by adopting both a within-network and a between-network approach from four-wave data among Chinese secondary school students. Our CFA results showed that whether the three domains were considered individually or together, the one-factor models (1, 3, 5, 7, and 8) failed to produce appropriate fit, whereas the two-factor models (2, 4, 6, 9, and 10) yielded superior fit, supporting the assumption that competence and affect should be conceptualized as two separate constructs among Chinese secondary school students.

### 5.1. The within-network evidence of the stability of the twofold multidimensional structure of ASC

Consistently with our first hypotheses, our study found that the stability of the twofold multidimensional structure of ASC was also valid with Chinese secondary school students. We found that both of the CFAs (model 7 and 8) which conflated competence and affect in the three academic domains generated non-fit, even when the negative items were eliminated (model 8), whereas both of the CFAs (model 9 and 10) which examined the stability of the validity of the twofold structure of ASC in the three domains produced good fit, even when the negative items were included (model 9). These results were consistent with the longitudinal study by Marsh et al. (1999b) with Australian primary school students. More recently, Leung (2019) also found strong support for the twofold multidimensional structure of the ASC among Chinese students, despite that Leung’s study was cross-sectional and used SDQ II (appropriate for the high school students). Our study together with studies by Leung and Yang et al. (2016) seemed to indicate that the twofold multidimensional structure of ASC was applicable for Chinese students who were above primary school levels.

Similar to the findings reported in previous cross-sectional studies (Arens et al., 2011; Abu-Hilal et al., 2013; Leung, 2019), we also observed that the competence scales were positively related to the affect scales within the Chinese, math, and general school domains, and such patterns were stable across the four-time points. Also aligned with previous cross-sectional studies (Arens et al., 2011; Yang et al., 2016; Schneider and Sparfeldt, 2020), we observed that the correlations between competence and affect in Chinese (*r*s ranging from 0.56 to 0.62) and math (*r*s ranging from 0.56 to 0.64) were consistently higher than those in general school (*r*s ranging from 0.37 to 0.42). These results may indicate that the relations between self-perceptions of cognitive ability and affective status in specific subjects are stronger than the relations between competence and affect in school learning in general and support the domain-specific nature of ASC.

The cross-domain correlations of the competence and affect components found in our study somewhat contradict the domain specificity of both the competence and affect components in Arens et al. (2011), as the cross-domain correlations of both the competence and affect components were negligible in their study. Our study only showed the domain specificity of competence components. The cross-domain relations between the affect components were moderate (*r*s ranging from 0.32 to 0.50), suggesting that students’ liking of the two dissimilar school subjects (Chinese and math) was not as pronounced as their own evaluation of their competence in these two subjects. One possible explanation could be that Chinese and math subjects, along with English, are the most highly valued subjects in the Chinese secondary school system, as the examination scores in these three subjects are given more weights than other subjects in the High School Entrance Examination. The affect developed in these two subjects, therefore, might not be based solely on students’ personal interests, but on the importance students attached to them.

### 5.2. The between-network evidence of the stability of the twofold multidimensional structure of ASC

Confirming the second hypothesis, our results showed consistently across the four-time waves that self-perceptions of competence were more strongly associated with achievement than self-perceptions of affect within both the Chinese and math domains. These results corroborated the findings of previous cross-sectional studies (Arens et al., 2011; Abu-Hilal et al., 2013; Lohbeck, 2019; Schneider and Sparfeldt, 2020) and longitudinal studies in a single domain (Pinxten et al., 2014; Arens et al., 2016; Han, 2019). The results also supported the assumptions made by Irwing (1996) that evaluation of one’s competence involves more external (social) comparison, whereas evaluation of one’s affect involves more internal (dimensional) comparison.

In our study across the Chinese and math domains, the relations between competence and achievement were much stronger for matching domains than those for non-matching domains in all the four waves, confirming the domain-specific nature of academic self-concept and the I/E model and the dimensional comparison model of self-concept (Marsh, 1986; Möller and Marsh, 2013). The magnitude of correlations between affect and achievement was also stronger within one domain than across domains for both Chinese and math learning except for wave three where we observed that the correlation between Chinese affect and Chinese achievement (*r* = 0.09) was slightly weaker than math affect and Chinese achievement (*r* = 0.10). Comparing the associations between achievement scores and affect in both matching and non-matching domains, the features of domain specificity which were normally found in the relations between achievement and competence were also identified in the relations between achievement and affect.

Stronger relations between competence and achievement than between affect and achievement were also observed between the Chinese and math achievement scores and the two components of ASC in general school. We found that for all four-time waves, Chinese and math achievement scores had stronger correlations with general school competence than with general school affect. Furthermore, the associations between general school competence and achievement scores in Chinese were similar to those between general school competence and achievement scores in math; this was also the case for the associations between general school affect and achievement scores in Chinese and math (except for wave three). These relational patterns between domain-specific achievements and general school competence and affect corroborated the findings in cross-sectional studies (Arens et al., 2011) and provided longitudinal evidence of the claim postulated in the Marsh/Shavelson model (Marsh, 1990d) that general school ASC incorporates both the verbal and math domains.

Although the relations of both components of ASC with achievements in non-matching domains were weaker than those found in the corresponding domains, they were neither zero nor negative at most time points, which did not seem to align with the predictions of the I/E model, and somewhat contradicted with our third hypothesis. A number of cross-sectional studies with younger learners (primary school students) also reported no zero or negative cross-domain relations between ASCs and achievements, which were inconsistent with the predictions of the I/E model (both competence and affect: Arens et al., 2011; Lohbeck, 2019; only competence: Ehm et al., 2014; Lohbeck and Möller, 2017). For example, both Lohbeck and Möller (2017) and Ehm et al. (2014) studies found positive and significant cross-domain relations in the sample of German 1st to 3rd graders. A number of possible explanations for such results were offered by these researchers, such as younger children’s less developed cognitive abilities with regard to dimensional comparison (Harter, 1999); younger learners were not as familiar with the grading system as they were at the beginning of their schooling (Lohbeck and Möller, 2017) or both contrast and assimilation processes were in operation (Möller and Marsh, 2013), in particular if the domains were less distinct (Ehm et al., 2014).

These explanations, however, might not be applicable to our study, as our participants were secondary students, and the verbal and math subjects were significantly different. One of the possible explanations is that our participants might engage in social comparison more than dimensional comparison, which might be attributable to three reasons: (1) the Chinese culture; (2) the developmental stage of the participants; and (3) the educational context of the participants. For the first reason, the Chinese culture is a collectivistic culture (Ho and Chiu, 1994; White and Lehman, 2005), and research has shown that people with higher level of collectivism tend to have a higher desire for social comparison (Chung and Mallery, 1999). White and Lehman (2005) argue that “those from Eastern and Western cultural contexts differ in the extent to which they construe the self as interconnected with or distinct from those around them.” As the proposal of the I/E model is largely based on earlier evidence garnered primarily from Western contexts, it may not be applicable to the Chinese culture. However, to confirm this conjecture, more empirical research evidence is required. Secondly, from the developmental perspective, our participants were in their secondary school students, who were in a period of re-orientating to the new learning contexts, being more sensitive to the social contexts and peer influence, and a significant increase in social interactions (Brown and Larson, 2009; Maehr and Zusho, 2009; Molloy et al., 2011; Crone and Dahl, 2012). Hence, our participants might have heightened engagement in social comparison. Last but not least, the predominance of social comparison over dimensional comparison might also be related to the Chinese educational context. In Chinese schools, academic social comparisons are a common phenomenon, in particular among Chinese adolescents (Fu et al., 2018; Li et al., 2021). In such an educational context, our participants might use more social comparison than dimensional comparison to rate their self-concepts in Chinese and math.

Other possible explanations of the unexpected results of the cross-domain correlations between both components of ASC and achievements could be some mediators, which had not been examined in our study. Recent studies have identified a range of factors could mediate between ASC and achievement, such as parental expectations (Phillipson and Phillipson, 2017), maternal control (Lu et al., 2017), and cultural norms (Wentzel et al., 2021; Vu et al., 2022). Hence, in order to fully understand the correlations between ASCs and achievements in non-matching domains, future studies should be designed by incorporating possible mediators. Despite these partial inconsistencies concerning the predictions of the I/E model, in general, our findings regarding the between-network approach also provided support for the validity of the twofold multidimensional nature of ASC in these groups of students across time.

### 5.3. Limitations and suggestions for future research

Some limitations of the study need to be pointed out so that they can be addressed in future studies. First, our sample size was not large considering that our study was longitudinal and involved many constructs. Moreover, the participants were recruited from the same secondary school. Although there were advantages of recruiting the students from the same school, such as their academic achievement results being comparable as the students sat the same examinations, this limited the generalization of the study results. Therefore, future studies should have a large sample size and recruit participants from different schools so that the sample would be more representative, which will provide stronger support for the study results. Second, our study only demonstrated the stability of the twofold multidimensional structure of ASC with Chinese secondary school students, future research should extend such longitudinal investigation into Chinese students of other age groups and students in other cultures. Another limitation is that our study did not include other important factors in learning, such as learning engagement (Henning et al., 2022), academic enjoyment (Liu et al., 2022), study interest (Fryer and Ainley, 2019), and achievement emotions (Forsblom et al., 2022). Including these important elements may help reveal complex relations (e.g., mediating relations) between ASC, learning outcomes, and these factors.

### 5.4. Conclusion

The current study examined the stability of the twofold multidimensional structure of ASC with Chinese secondary school students. It filled the gap in the literature where there was a lack of research examining the separation of competence and affect in multiple domains using both a within-network and a between-network approach. While the majority of past studies on the validity of the twofold multidimensional structure of ASC were conducted with learners from Western and Arab cultures, our study provided additional evidence in this area by including a less researched population – Chinese learners. Our results demonstrate that both the multidimensional and twofold nature of academic self-concept obtained from cross-sectional studies with Western samples is applicable to our sample of Chinese secondary students.

## Data availability statement

The raw data supporting the conclusions of this article will be made available by the authors, without undue reservation.

## Ethics statement

The studies involving human participants were reviewed and approved by the Human Research Ethics Committee of the University of Sydney. Written informed consent to participate in this study was provided by the participants’ legal guardian/next of kin.

## Author contributions

FH contributed substantially to the conception of the work, the acquisition, analysis, and interpretation of the data. FH, KJ, PM, and LN drafted the work and revised it critically for important intellectual content, approved the final version of the paper to be published, and agreed to be accountable for all aspects of the work in ensuring that questions related to the accuracy or integrity of any part of the work are appropriately investigated and resolved.

## Funding

This study was supported by the International mobilities for research activities of the University of Hradec Králové II, CZ.02.2.69/0.0/0.0/18_053/0017841; Faculty of Education, the University of Hradec Králové as part of the projects: Individual and contextual predictors of the effect of supporting academic autonomy in future teachers (number: 2101); the Competition for Postdoctoral Job Positions (number: 2201); and Excessive use of social networks and emotional intelligence (number: 2110).

## Conflict of interest

The authors declare that the research was conducted in the absence of any commercial or financial relationships that could be construed as a potential conflict of interest.

## Publisher’s note

All claims expressed in this article are solely those of the authors and do not necessarily represent those of their affiliated organizations, or those of the publisher, the editors and the reviewers. Any product that may be evaluated in this article, or claim that may be made by its manufacturer, is not guaranteed or endorsed by the publisher.
